# Dexamethasone increased the survival rate in *Plasmodium berghei*-infected mice

**DOI:** 10.1038/s41598-021-82032-7

**Published:** 2021-01-29

**Authors:** Danilo Reymão Moreira, Ana Carolina Musa Gonçalves Uberti, Antonio Rafael Quadros Gomes, Michelli Erica Souza Ferreira, Aline da Silva Barbosa, Everton Luiz Pompeu Varela, Maria Fani Dolabela, Sandro Percário

**Affiliations:** 1 Oxidative Stress Research Laboratory, Institute of Biological Sciences, Federal University of Pará, Av. Augusto Corrêa, 01, Belém, PA 66075-110 Brazil; 2 Laboratory of Pathophysiology and Therapeutic Research, Centro de Ciências Sociais Saúde e Tecnologia – CCSST, Federal University of Maranhão, Campus Avançado - Bom Jesus, Prédio de Medicina, Av. da Universidade, S/N, Imperatriz, MA 65915-240 Brazil; 3 Institute of Health Sciences, Federal University of Pará, Av. Augusto Corrêa, 01, Belém, PA 66075-110 Brazil

**Keywords:** Infectious diseases, Parasitology

## Abstract

The present study aimed to evaluate the effects of dexamethasone on the redox status, parasitemia evolution, and survival rate of *Plasmodium berghei*-infected mice. Two-hundred and twenty-five mice were infected with *Plasmodium berghei* and subjected to stimulation or inhibition of NO synthesis. The stimulation of NO synthesis was performed through the administration of L-arginine, while its inhibition was made by the administration of dexamethasone. Inducible NO synthase (iNOS) inhibition by dexamethasone promoted an increase in the survival rate of *P*. *berghei*-infected mice, and the data suggested the participation of oxidative stress in the brain as a result of plasmodial infection, as well as the inhibition of brain NO synthesis, which promoted the survival rate of almost 90% of the animals until the 15th day of infection, with possible direct interference of ischemia and reperfusion syndrome, as seen by increased levels of uric acid. Inhibition of brain iNOS by dexamethasone caused a decrease in parasitemia and increased the survival rate of infected animals, suggesting that NO synthesis may stimulate a series of compensatory redox effects that, if overstimulated, may be responsible for the onset of severe forms of malaria.

## Introduction

According to the World Health Organization (WHO), malaria is a significant public health problem in 97 countries and causes approximately 219 million new cases each year, resulting in 435,000 deaths in 2017^1^.

In this regard, several authors recently discussed the involvement of free radicals in the physiopathogenesis of malaria. This involvement can be related to the pathogenic mechanisms triggered by the parasite^2^, as well as by the production of free radicals and antioxidant defenses by host cells as an attempt to fight the infection^3,4^.

In parallel, the relationship between the redox state of the parasite and host cells is overly complex and involves the production of nitric oxide (NO)^5^, which seems to play an important role. However, while some authors suggest that inhalation of this gas is a potential tool for the treatment of these complications^6,7^, others blame nitric oxide synthesis as responsible for causing respiratory distress syndrome^8^, in particular as a result of the activation of iNOS^9,10^.

NO acts as a key molecule in brain infections. It is still unknown whether the major problem arises from insufficient concentrations of NO^11^ acting directly in the elimination of the parasite, and for this reason, by selecting more resistant strains of the parasite, or from the high concentrations of NO produced as a result of infection by the protozoan parasite and responsible for cerebral edema^12,13^.

In fact, some researchers suggest a protective role of nitric oxide in the development of severe malaria and indicate it as a possible adjuvant in malaria drug therapy^14,15^. As suggested by Planche et al.^16^, the activation of NOS II is essential for the additional production of NO and elimination of the parasite. Notwithstanding, low NO bioavailability is associated with further development of cerebral malaria in mice models of the disease, possibly by eNOS/nNOS uncoupling, as a consequence of oxidative stress^17^.

The administration of L-arginine has been employed to stimulate the activity of iNOS in several studies, yet with controversial results^3^. On the other hand, NOS enzymes can be selectively inhibited. Among the most commonly used inhibitors, N-nitro-L-arginine methyl ester (L-NAME) and N-monomethyl-L-arginine (L-NMMA) inhibit both forms of the enzyme, while aminoguanidine and dexamethasone selectively inhibit iNOS^18^.

Previous studies have demonstrated the ability of dexamethasone to inhibit iNOS expression in various types of cells^19–24^. De Vera et al.^25^ attributed this action of dexamethasone to the inhibition of NF-κB and to the activation of its inhibitory factor (IFκB). Administering dexamethasone to *P*. *berghei*-infected mice significantly reduces symptoms of cerebral malaria^26,27^. Thus, the present study aims to verify the effects of iNOS-derived NO synthesis on oxidative markers and on the progression of parasitemia in *P*. *berghei*-infected mice, as well as on the survival rate of the animals.

## Material and methods

### Animals

Two-hundred and seventy-five male Swiss mice (*Mus musculus*), young adults (25–35 g; 6–8 weeks) from the Evandro Chagas Institute (Belem, PA, Brazil) were randomly divided into four groups, each of which was further divided into five subgroups, according to the time of animal euthanasia (one, five, ten, fifteen or twenty days after inoculation), by simple randomization generated after sortition^28^.

#### Positive control groups (PC; N = 15 for each subgroup)

Animals were inoculated with *P*. *berghei*-infected erythrocytes and received 10 µl of sterile distilled water per 25 g of body weight (gavage) two hours prior to the inoculation of *P*. *berghei*, and daily until the day of animal euthanasia.

#### Dexamethasone groups (N = 15 for each subgroup)

Animals were inoculated with *P*. *berghei* in the same way that PC groups and treated with dexamethasone (095214; TEUTO – Anapolis – GO—Brazil; *i*.*p*. *;* 5 mg kg^−1^ of animal weight) until the day of animal euthanasia.

#### L-arginine groups (N = 15 for each subgroup)

Animals were inoculated with *P*. *berghei* in the same way as PC groups and simultaneously treated with L-arginine (A5006; SIGMA ALDRICH – St. Louis – USA; 120 mg kg^−1^ of animal weight through gavage) until the day of animal euthanasia^29^.

#### Negative control groups (NC; N = 10 for each subgroup)

Animals were manipulated in the same way as PC groups, but with the inoculation of uninfected erythrocytes, and received 10 µl of sterile distilled water per 25 g of body weight (gavage) two hours prior to the inoculation of erythrocytes and daily until the day of animal euthanasia.

Both dexamethasone and L-arginine were administered 24 h prior to infection and every 24 h henceforth until the day of animal euthanasia.

All animals were maintained in the vivarium at the Federal University of Pará (UFPA, Belem, PA, Brazil) in polystyrene cages containing five animals each, kept under 12 h light/dark cycles, controlled temperature (25 °C), and received rodent chow (Labina, PRESENCE, Brazil) and tap water ad libitum for one, five, ten, fifteen or twenty days after infection and, at the end of each period, animals were submitted to heparin administration (100 UI heparin sulfate, *i*.*p*.), anesthetized with 50 μl of intraperitoneal ketamine (125 mg kg^−1^)-xylazine (25 mg kg^−1^), sample collection, and euthanasia by exsanguination.

After thoracotomy, blood samples were obtained by cardiac puncture of the right ventricle, and both lungs and brain were removed. The project followed the international guidelines for research with experimental animals and adhere to the ARRIVE guidelines for the reporting of animal experiments. Procedures were reviewed and approved by the Ethics Committee in Research with Experimental Animals of the Federal University of Pará—CEPAE/UFPA (Report No. MED0126/2013).

### Features of the animal model

Swiss mice are widely used as a malaria model and present the same pattern of infection progression and basic features of lung and cerebral malarias of other mouse species. Generally, 50% of these mice present clinical cerebral malaria on days 6–9 post-infection, although some develop it later (around days 15–20)^30^. Moreover, *P*. *berghei* possesses genomic sequences similar to those of *P*. *falciparum*^31^ and causes clinical features in animals that mimic human falciparum malaria^32,33^. Taken together, the histopathological features described are similar to those displayed in severe malaria human cases.

### Malaria induction

Mice were kept in the vivarium for two weeks and underwent clinical examination prior to malaria induction through intraperitoneal inoculation of 10^6^ *P*. *berghei* ANKA-infected erythrocytes (in 0.2 mL sterile saline solution). The strain of *P*. *berghei* was supplied by the Neurochemistry Laboratory of the Federal University of Pará—UFPA and replicated three times in Swiss mice before being used in the animals of this study.

### Determination of parasitemia

*Plasmodium berghei*-infected erythrocytes were counted on blood smears obtained by puncture of the caudal vein of animals on the day of euthanasia (one, five, ten, fifteen, and twenty days of infection). After drying at room temperature (25 °C), smears were fixed with methanol for 2 min and stained with Giemsa for 10 min. Subsequently, slides were washed in tap water, and after drying, infected erythrocytes were counted on an optical microscope (OLYMPUS, CX2) with 100 × magnification.

### Tissue processing

After removal, the lungs and brain were perfused with phosphate buffered saline (PBS) to wash out the blood trapped inside. The tissue was weighed and added to PBS at a ratio of 1:10 (m:v) and homogenized in an ultrasonic cell disruptor (D Cel; THORNTON, Indaiatuba, Brazil). During the process, the glass beaker containing the material was kept on ice to prevent sample damage. The homogenate was centrifuged at 175 × *g* (15 min), and the supernatant was collected and stored in a freezer at − 20 °C until analysis.

### Technical procedure

Along with blood parasitemia determination, laboratory measurements of oxidative stress markers were performed in duplicate on tissue samples. Internal controls and standards were inserted in each batch for the quality assurance of determinations.

#### Determination of Trolox Equivalent Antioxidant Capacity (TEAC)

Trolox (6-hydroxy-2,5,7,8-tetramethylchromane-2-carboxylic acid; SIGMA-ALDRICH 23881-3) is a powerful antioxidant water-soluble vitamin E analogue. The method proposed by Re et al.^34^ was followed, a colorimetric technique based on the reaction between ABTS (2,2′-azino-bis-3-ethylbenzothiazoline-6-sulfonic acid; SIGMA-ALDRICH; 1888) with ammonium persulfate potassium (K_2_S_2_O_8_; SIGMA-ALDRICH; 60490), producing the radical cation ABTS^●+^, chromophore of green/blue color. The addition of antioxidants to ABTS^●+^ reduces it again to ABTS on a scale dependent on antioxidant capacity, concentration of antioxidants and duration of the reaction. This can be measured by spectrophotometry by observing the change in absorbance read at 734 nm for five minutes (FENTO, Sao Paulo, Brazil; 800 XI). Finally, the total antioxidant activity of the sample is calculated as its relationship with the reactivity of Trolox as a standard through the implementation of a standard curve under the same conditions.

#### Determination of Thiobarbituric Acid Reactive Substances (TBARS)

TBARS is a method that evaluates lipid peroxidation and was used as an indicator of oxidative stress. This technique is based on the reaction of malondialdehyde (MDA), among other substances, with thiobarbituric acid (TBA; SIGMA-ALDRICH, T5500) at low pH and high temperature, yielding a pink MDA-TBA complex with an absorbance peak at 535 nm. The technical procedure was performed according to the protocol adapted by Percário et al.^35^. Samples were collected and read at 535 nm (FENTO, São Paulo, Brazil; 800 XI). 1,1,3,3, tetraethoxypropane (SIGMA-ALDRICH; T9889) was used for the implementation of the standard curve.

#### Nitrites and nitrates (NN)

Much of the nitric oxide released into the bloodstream is swept by hemoglobin in erythrocytes or converted to nitrite (NO_2_^●−^) in the presence of molecular oxygen. Nitrite reacts with oxyhemoglobin, leading to the formation of nitrate (NO_3_^●−^) and methemoglobin. Due to its stability, NO_2_^●−^ has been widely used to confirm the prior existence of NO. The evaluation of this parameter was performed by means of spectrophotometry (Kit Total Nitrite/Nitrate, R & D SYSTEMS, KGE001). This technique is based on the quantitative determination of NO, involving the enzyme nitrate reductase, which converts nitrate to nitrite, followed by colorimetric detection of nitrite as a product of pink color, produced by the Griess reaction and that absorbs visible light at 540 nm (PERKIN-ELMER, Victor X3). The nitrite concentration was calculated based on the absorbance found in the nitrite standard curve^5^.

#### Uric acid (AU)

Performed using the Uric acid UOD-ANA Kit (LABTEST, Cat. 51-4/30). Samples were read at 520 nm using a spectrophotometer (BIOSPECTRO, SP-22, Brazil).

### Statistical analysis

Aiming at investigating the existence of statistically significant differences between the studied variables between groups, we applied two factor ANOVA, when the assumption of normality and homoscedasticity was met, or the Mann–Whitney test, when the assumption of normality was not met, which occurred in the case of variable PARASITEMIA. When the null hypothesis between mean differences between the variables of the study groups was rejected, Tukey's test was applied, and when a statistically significant difference between medians was detected, Dunn’s test was applied. In addition, within the same group, the differences between the initial values (1 day of infection) and late values (20 days of infection) were studied by Student’s unpaired t test.

The existence of correlation between the variables was also analyzed by Pearson's correlation coefficient, considering all points obtained separately for each group studied. For the statistically significant correlations, intensities were assigned as follows: r up to 0.30 (r < 0.30) as weak correlation; r between 0.31 and 0.70 (0.31 < r < 0.70) as moderate correlation; r between 0.71 and 1.00 (0.71 < r < 1.00) as strong correlation.

For the purposes of ANOVA and Mann–Whitney tests, the statistical package SigmaStat version 3.5 was used, whereas for the calculation of correlations, the statistical package SPSS version 17.0 was used. All statistical tests were applied considering the significance level of 5% (*p* < 0.05).

### Ethics approval

The project followed the international guidelines for research with experimental animals, and procedures were reviewed and approved by the Ethics Committee in Research with Experimental Animals of the Federal University of Pará—CEPAE/UFPA (Report No. MED0126/2013).

## Results

**Figure 1:**
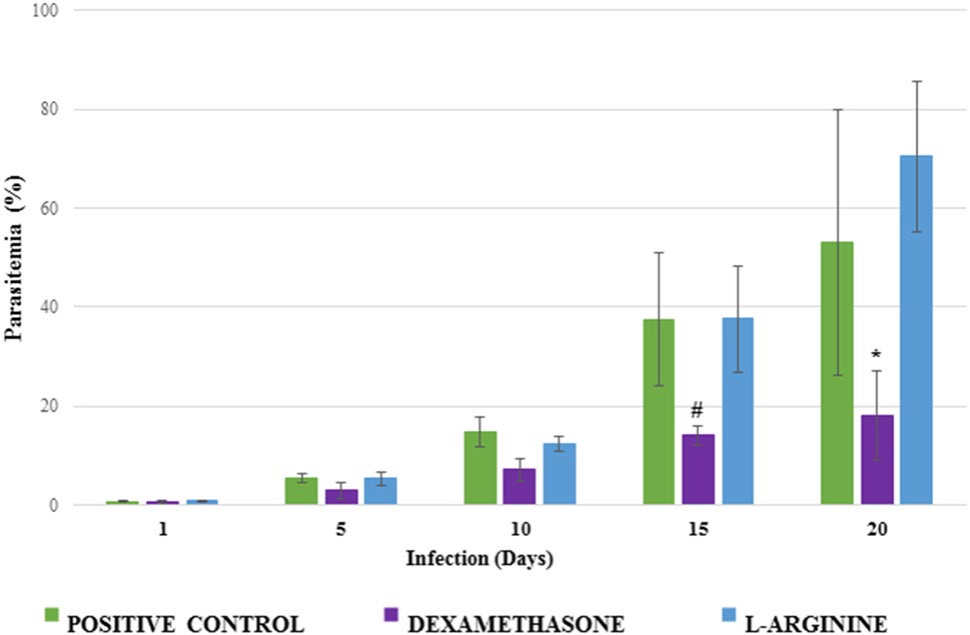
Progression of parasitemia in *Plasmodium berghei*-infected Swiss mice. Animals were pretreated and received a daily dose of DEXAMETHASONE, L-ARGININE, or PBS (CONTROL). ^#^p = 6.8 × 10^−6^ versus L-ARGININE and p = 7.3 × 10^−5^ versus CONTROL; *p = 2.8 × 10^−5^ versus L-ARGININE and p = 0.0227 versus CONTROL.

As expected, parasitemia of infected animals progressively evolved in all groups, but the rate of progression was lower in dexamethasone-treated animals, which presented lower parasitemia values than the other two groups at the end of the period of 20 days (p = 2.8 × 10^−5^ vs. L-arginine and p = 0.0227 vs. control; Fig. 1). L-arginine-treated animals presented numerically higher values than the control group, but without statistical significance (p = 0.3048).

**Figure 2:**
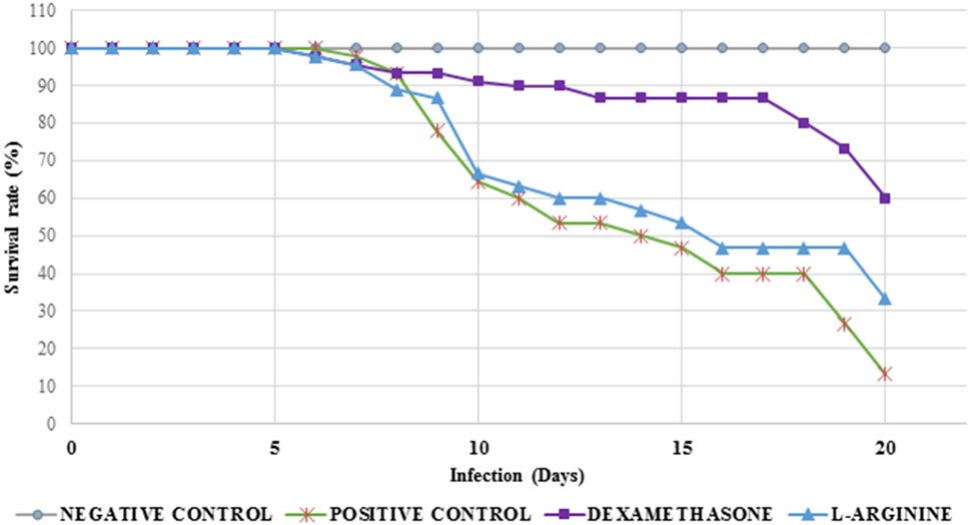
Survival rate of *Plasmodium berghei*-infected Swiss mice. Animals were pretreated and received a daily dose of DEXAMETHASONE, L-ARGININE, or PBS (CONTROL).

Similarly, the survival rate of dexamethasone-treated animals was significantly greater than that of the other groups, which behaved in a similar way, with 60% of animals alive at the end of the period of 20 days of infection (p = 0.00548 vs. L-arginine and p = 0.00386 vs. control; Fig. 2).

### Determination of Trolox equivalent antioxidant capacity

**Figure 3:**
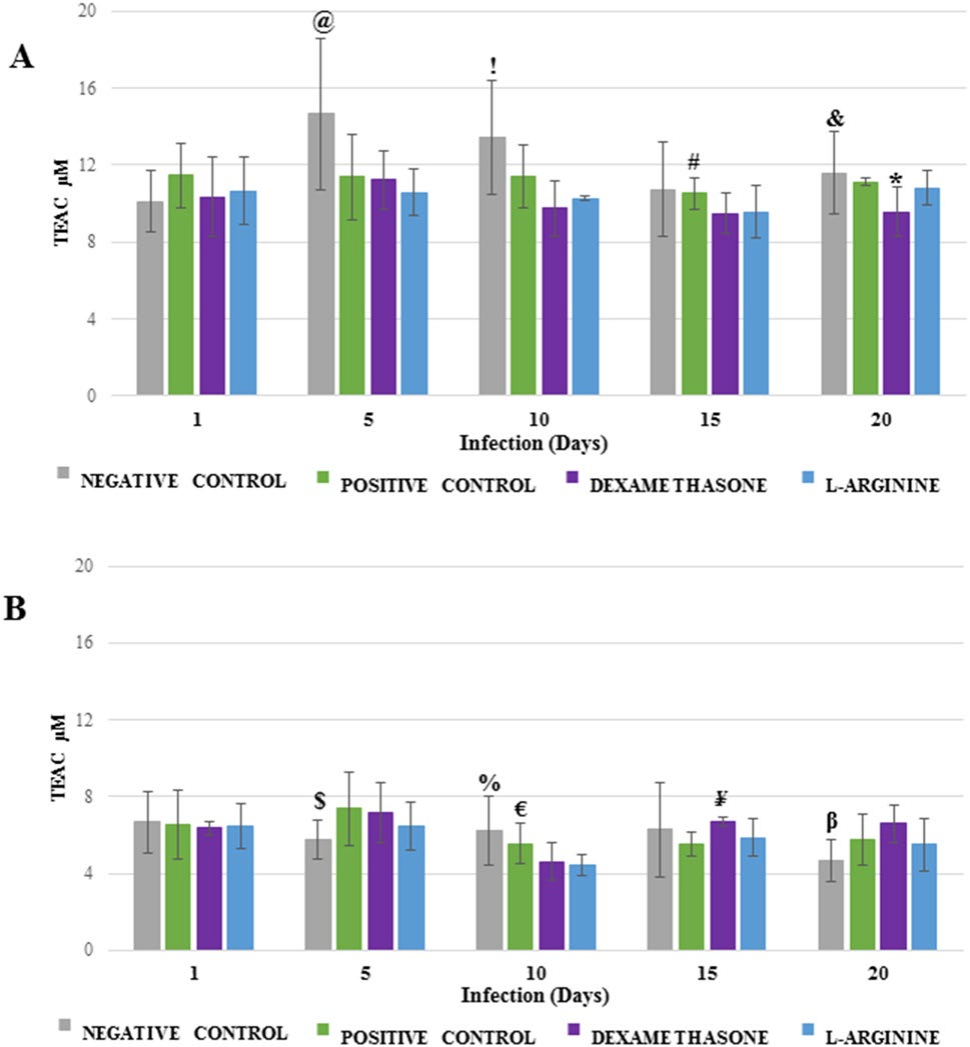
Trolox equivalent antioxidant capacity (TEAC) in the lungs (**A**) and brains (**B**) of *Plasmodium berghei*-infected Swiss mice. Animals were pretreated and received a daily dose of DEXAMETHASONE, L-ARGININE, or PBS (CONTROL). ^@^p = 0.0145 versus POSITIVE CONTROL, p = 0.0057 versus DEXAMETHASONE, and p = 0.0009 versus L-ARGININE; ^!^p = 0.0011 versus DEXAMETHASONE and p = 0.0215 versus L-ARGININE; ^#^p = 0.0401 versus DEXAMETHASONE; ^&^p = 0.0247 versus DEXAMETHASONE; *p = 0.0281 versus L-ARGININE and p = 0.0033 versus POSITIVE CONTROL; ^$^p = 0.0286 versus POSITIVE CONTROL and p = 0.0273 versus DEXAMETHASONE; ^%^p = 0.0160 versus DEXAMETHASONE; ^€^p = 0.0360 versus L-ARGININE and p = 0.0261 versus DEXAMETHASONE; ^¥^p = 0.0357 versus L-ARGININE and p = 0.0005 versus POSITIVE CONTROL; ^β^p = 0.0010 versus DEXAMETHASONE.

For the lung samples, there were no statistically significant differences in TEAC values during the period of infection (Fig. 3A). Nevertheless, at the end of 20 days of infection, the group of animals treated with dexamethasone presented statistically lower values than the other two groups (p = 0.0281 vs. L-ARGININE and p = 0.0033 vs. POSITIVE CONTROL). For brain samples, a similar behavior was observed; however, an important decrease in TEAC after 10 days of infection was identified (1 day vs. 10 days, p = 0.0009 for L-ARGININE and p = 7 × 10^−6^ for DEXAMETHASONE; Fig. 3B), with both treated groups presenting values lower than the control group (p = 0.0360 vs. L-ARGININE and p = 0.0261 vs. DEXAMETHASONE; Fig. 3B). However, after the 10th day of infection, the DEXAMETHASONE group presented an increase in TEAC values, displaying statistically higher values than the other groups (p = 0.0357 vs. L-ARGININE and p = 0.0005 vs. POSITIVE CONTROL: Fig. 3B).

### Determination of thiobarbituric acid reactive substances

**Figure 4:**
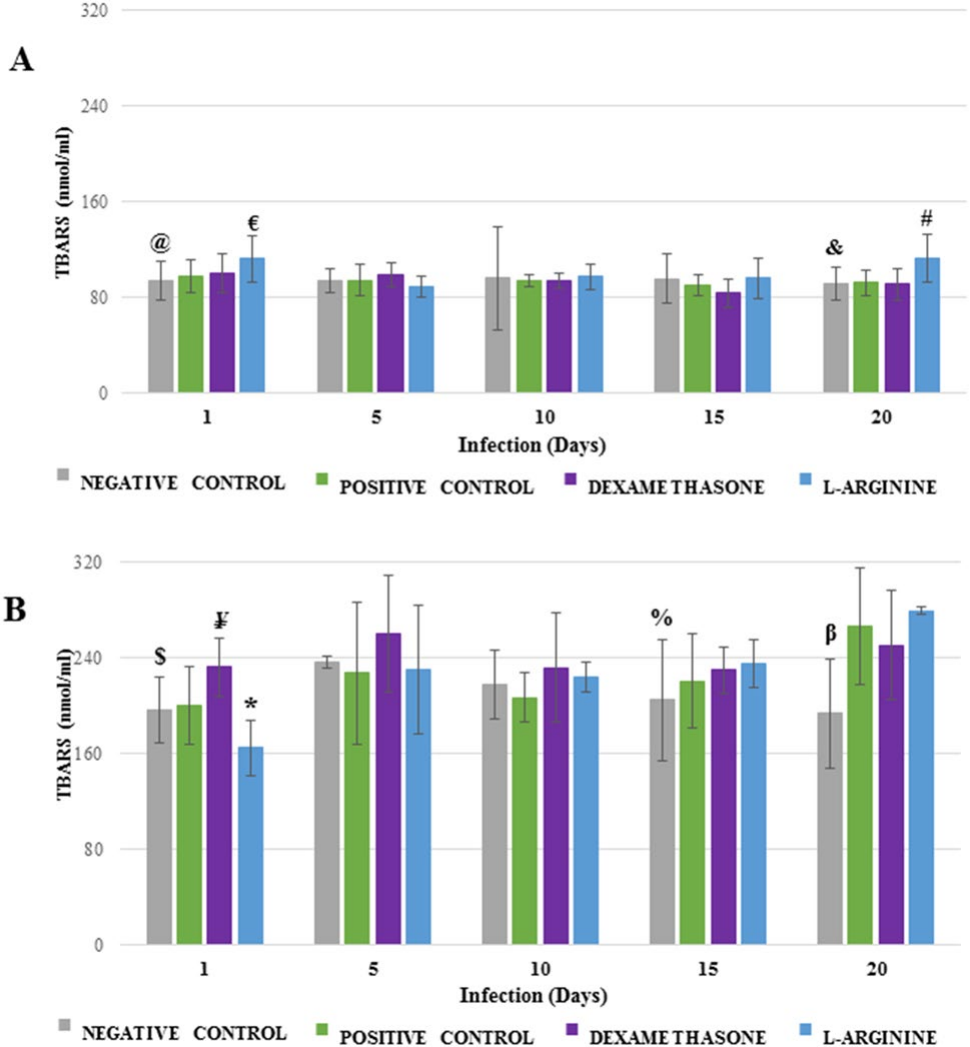
Thiobarbituric acid reactive substances (TBARS) in the lungs (**A**) and brains (**B**) of *Plasmodium berghei*-infected Swiss mice. Animals were pretreated and received a daily dose of DEXAMETHASONE, L-ARGININE, or PBS (CONTROL). ^@^p = 0.0212 versus L-ARGININE; ^€^p = 0.0294 versus POSITIVE CONTROL; ^&^p = 0.0304 versus L-ARGININE; ^#^p = 0.0282 versus DEXAMETHASONE; ^$^p = 0.0023 versus DEXAMETHASONE; ^¥^p = 0.0060 versus POSITIVE CONTROL; *p = 0.0005 versus DEXAMETHASONE and p = 0.0029 versus POSITIVE CONTROL; ^%^p = 0.4106 versus L-ARGININE; ^β^p = 0.0175 versus DEXAMETHASONE and p = 0.0.0169 L-ARGININE.

Although none of the groups presented important variation during the period of infection, L-ARGININE group presented higher pulmonary TBARS values than the DEXAMETHASONE group at the end of the experiment (p = 0.0282; Fig. 4A). On the other hand, for brain samples, L-ARGININE group showed progressive evolution over the period of the infection, with higher TBARS values on the 20th day of infection in relation to the first day (p = 4.7 × 10^−7^), but with no differences in relation to the other groups (Fig. 4B).

The collective analysis of the values of TEAC and TBARS shows a quite unique pattern: while for lung samples, the values of TEAC obtained are found in a high range of absolute values (9–12 µM), brain samples are in a low range (4–8 µM), whereas TBARS values present opposing behavior, *i*.*e*. for lung samples, the values are in the low range (80–120 nmol mL^−1^), and brain samples are in the high range (160–280 nmol mL^−1^).

### Nitrites and nitrates

**Figure 5:**
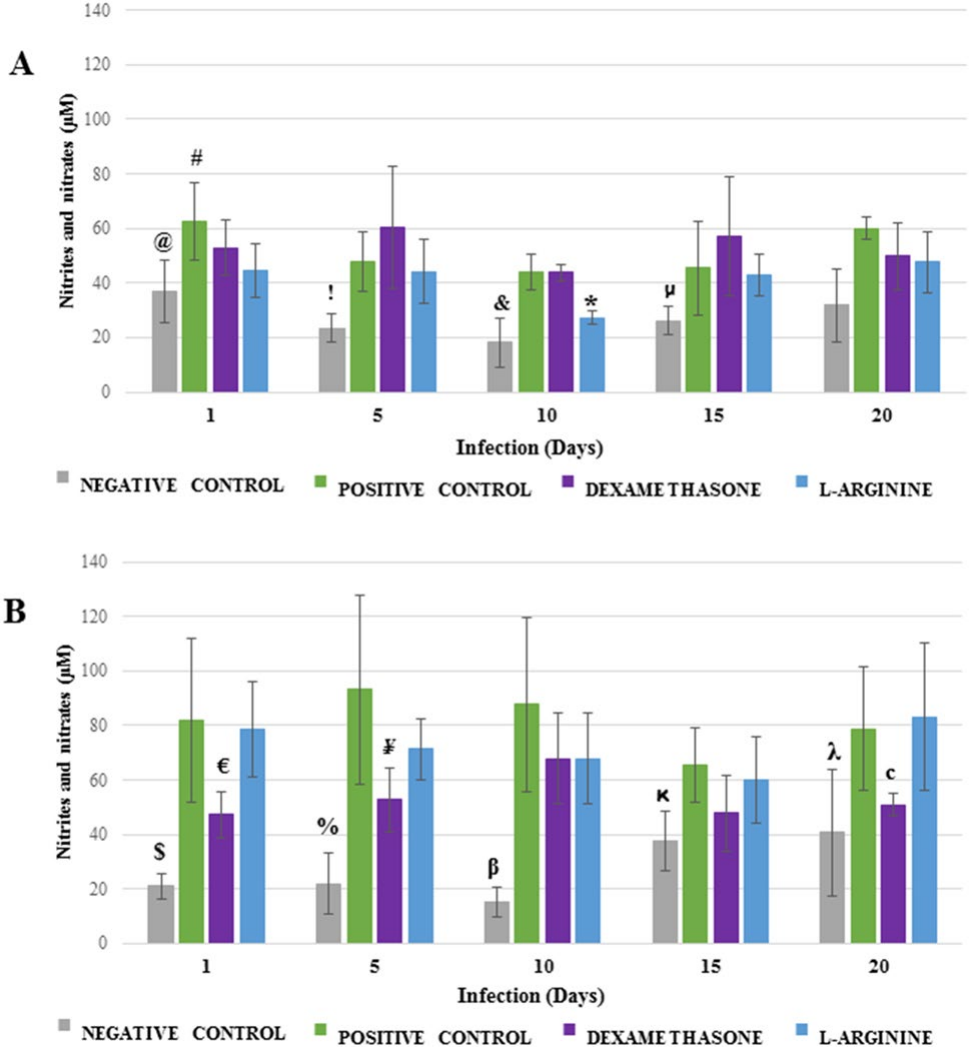
Nitrites and nitrates in the lungs (**A**) and brains (**B**) of *Plasmodium berghei*-infected Swiss mice. Animals were pretreated and received a daily dose of DEXAMETHASONE, L-ARGININE, or PBS (CONTROL). ^@^p < 0.0001 versus POSITIVE CONTROL and p = 0.0013 versus DEXMETHASONE; ^#^p = 0.0005 versus L-ARGININE and p = 0.0394 versus DEXAMETHASONE; ^!^p = 0.0006 versus POSITIVE CONTROL and p < 0.0001 versus DEXAMETHASONE and p < 0.0001 versus L-ARGININE; ^&^p < 0.0001 versus POSITIVE CONTROL and p < 0.0001 versus DEXAMETHASONE and p = 0.0158 versus L-ARGININE; *p = 3.1 × 10^−5^ versus DEXAMETHASONE and p = 1.5 × 10^−4^ versus POSITIVE CONTROL; ^μ^p = 0.0048 versus POSITIVE CONTROL and p = 0.0003 versus DEXAMETHASONE and p = 0.0001 versus L-ARGININE; ^$^p < 0.0001 versus POSITIVE CONTROL and p < 0.00001 versus L-ARGININE; ^€^p = 3.4 × 10^−6^ versus L-ARGININE and p = 5.0 × 10^−4^ versus CONTROL; ^%^p < 0.00001 versus POSITIVE CONTROL and p < 0.00001 versus DEMATHASONE and p = 0.0001 versus L-ARGININE; ^¥^p = 3.6 × 10^−4^ versus L-ARGININE and p = 4.6 × 10^−4^ versus POSITIVE CONTROL; ^β^p = 0.0007 versus POSITIVE CONTROL, p = 0.0007 versus DEXAMETHASONE, and p = 0.0057 versus L-ARGININE; ^κ^p = 0.0001 versus POSITIVE CONTROL and p = 0.0015 versus L-ARGININE; ^λ^p = 0.0094 versus L-ARGININE; ^c^p = 0.0058 versus L-ARGININE and p = 0.0201 versus POSITIVE CONTROL.

Despite displaying higher levels than the NEGATIVE CONTROL group, no significant differences in the temporal evolution of NN levels in any of the infected groups throughout the period of infection were observed, nor between infected groups for the lung samples (Fig. 5). However, for brain samples, DEXAMETHASONE group presented lower values than the other two groups during the studied period, culminating with statistically significant differences on the 20th day (p = 0.0058 vs. L-ARGININE and p = 0.0201 vs. POSITIVE CONTROL).

### Uric acid

**Figure 6:**
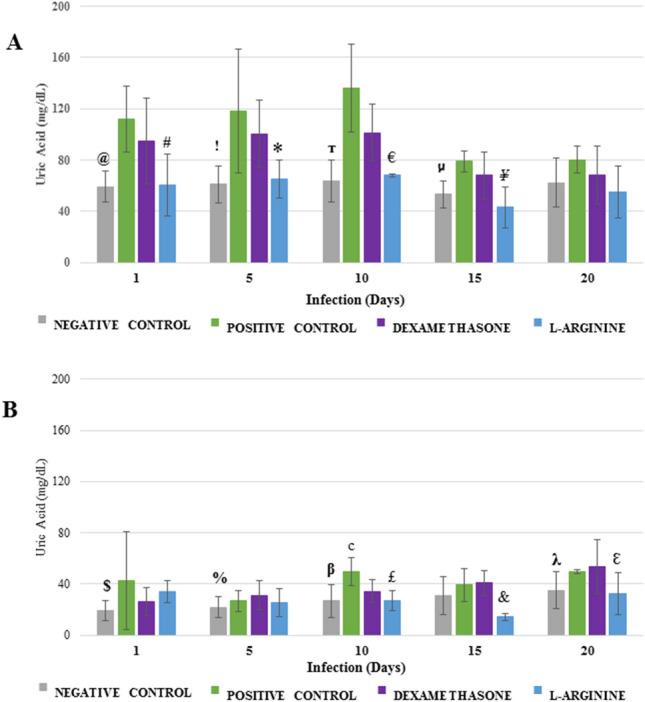
Uric acid levels in the lungs (**A**) and brains (**B**) of *Plasmodium berghei*-infected Swiss mice. Animals were pretreated and received a daily dose of DEXAMETHASONE, L-ARGININE, or PBS (CONTROL). ^@^p < 0.0001 versus POSITIVE CONTROL and p = 0.0013 versus DEXAMETHASONE; ^#^p = 0.0033 versus DEXAMETHASONE and p = 4.3 × 10^−6^ versus POSITIVE CONTROL; ^!^p = 0.0016 versus POSITIVE CONTROL and p = 0.0003 versus DEXAMETHASONE; *p = 0.00058 versus POSITIVE CONTROL; ^т^p < 0.0001 versus POSITIVE CONTROL and p = 0.0002 versus DEXAMETHASONE; ^€^p = 0.00095 versus POSITIVE CONTROL and p = 0.00054 versus DEXAMETHASONE; ^μ^p = 0.0001 versus POSITIVE CONTROL and p = 0.0139 versus DEXAMETHASONE; ^¥^p = 0.00071 versus POSITIVE CONTROL and p = 0.01167 versus DEXAMETHASONE; ^$^p = 0.0004 versus L-ARGININE; ^c^p = 0.0080 versus DEXAMETHASONE and p = 0.0024 versus L-ARGININE; ^%^p = 0.0426 versus DEXAMETHASONE; ^β^p = 0.0032 versus POSITIVE CONTROL; ^£^p = 0.0479 versus DEXAMETHASONE; ^&^p = 0.0029 versus POSITIVE CONTROL and p = 0.0001 versus DEXAMETHASONE; ^λ^p = 0.0398 versus L-ARGININE; ^ε^p = 0.0395 versus POSITIVE CONTROL and p = 0.0407 versus DEXAMETHASONE.

No temporal variation in AU values for lung samples in any of the groups was found (Fig. 6). However, L-ARGININE group presented lower values than the other two groups from the first to the 15th day of infection (Fig. 6A). Similarly, for brain samples (Fig. 6B), L-ARGININE group presented lower values than the other groups, with statistical significance at the 20th day of infection (p = 0.0395 vs. POSITIVE CONTROL and p = 0.0407 vs. DEXAMETHASONE). In contrast, DEXAMETHASONE group presented progressive behavior over the infection time, with brain AU values significantly greater for the 20th day in comparison to the first day (p = 3.9 × 10^−4^). Another noteworthy observation is that pulmonary AU values stood in a higher range (40–140 mg dL^−1^) than the brain AU (10–55 mg dL^−1^) for all groups.

### Correlation studies

#### Parasitemia versus TBARS

The correlation between TBARS and PARASITEMIA revealed the existence of a negative and significant correlation only for the lung samples from the DEXAMETHASONE group (Additional file 1 Figure S1; r = − 0.29; p = 0.026). The POSITIVE CONTROL group presented a negative correlation, however, without statistical significance (r = − 0.10; p = 0.20), while for the L-ARGININE group, this correlation showed positive but non-significant values (r = 0.06; p = 0.673). For brain samples, a positive trend was observed for all groups, but only with significance for group L-ARGININE (Additional file 1 Figure S2; r = 0.46; p = 0.002).

#### TBARS versus uric acid

A positive correlation was observed for these parameters in both samples and for group POSITIVE CONTROL (Additional file 1 Figures S3–S4; r = 0.33 and p = 0.02, for lung; r = 0.45 and p = 0.050, for brain) and DEXAMETHASONE (r = 0.26 and p = 0.041, for lung; r = 0.28 and p = 0.045, for brain). For the L-ARGININE group, in both samples, the values of the coefficient of correlation approached zero (r = 0.08 and p = 0.140, for lung; r = 0.03 and p = 0.858, for brain).

#### NN versus TBARS

For lung samples, the existence of a significant correlation for any of the studied groups was not observed (Additional file 1 Figure S5). However, for brain samples, both POSITIVE CONTROL and DEXAMETHASONE groups presented significant positive correlations (Additional file 1 Figure S6; r = 0.30 and p = 0.048 and r = 0.34 and p = 0.014, respectively).

### Other correlations

In addition to the studies of correlation presented, we tested several other correlations, but with no significance (Additional file 1 Figures S7–S20).

## Discussion

In this study, the treatment with glucocorticoid dexamethasone pointed to a significant increase in the percentage of survival rate for the groups of mice in comparison to other groups. This seems to be correlated with the evolution of parasitemia in these animals, which changed less in this group and remained, from the 5th day henceforth, significantly lower than the other groups.

It is important to highlight that dexamethasone is a steroidal anti-inflammatory drug that acts through iNOS mRNA synthesis inhibition^36^, and presents several immunological actions, such as tumor necrosis factor-α (TNF-α) inhibition^37^, and the inhibition of cachectin production and immunological mediators in the hamster cheek model^38^. Nevertheless, dexamethasone differentially affects several gene clusters, particularly inhibiting a large cluster of interferon γ (IFN-γ) induced genes^39^.

Notwithstanding, the effect of dexamethasone on the evolution of parasitemia can promote the inhibition of oxidative stress, as may be suggested by the existence of a negative correlation between TBARS and PARASITEMIA found only for the animals in the DEXAMETHASONE group. Contrarily, Rungruang and Klosek^40^ treated *P*. *yoelli*-infected mice with dexamethasone and found increased development and maturation of parasites. However, the dose employed was very low (0.5 mg kg^−1^) in comparison to the dose employed in the present study (5 mg kg^−1^).

In the same way, the effect of L-arginine is consistent with the effect found in this correlation for the animals of the L-ARGININE group, where there is the reversal of the pattern displayed by the DEXAMETASONE group, presenting positive values of correlation.

Contrary to that mentioned by some authors^14,15,29,41^ that attribute a protective role to nitric oxide in malaria, mice treated with L-arginine remained with a percentage of parasitemia and survival rate comparable to the POSITIVE CONTROL group, suggesting that NO synthesis is not involved among the initial mechanisms of host defense and, therefore, may not contribute to the elimination of parasites. However, the mentioned studies have measured the survival rate of animals treated with dipropylene triamine NONOate, a natural donor of nitric oxide, active in acid PH (common in malaria) whose action, unlike L-arginine, is independent of enzyme activation. Notwithstanding, most of NO effects were attributed to its action on the circulatory system of those animals. Another study with mice treated with L-arginine found no improvement on survival rate and parasitemia, despite increased NO biodisponibility^42^.

Nevertheless, plasmodial infection causes arginine depletion in mice and children with cerebral malaria, and plasma arginase consumption by NOS is reduced in infected mice. These findings suggest that arginine depletion derives from a decreased rate of appearance, rather than an increased consumption by NOS^43^.

### Pulmonary findings

In the present study, there was no significant variation in the dosages of TEAC and TBARS levels in the control group during the infection period. However, a positive correlation between these parameters was observed for both samples tested, suggesting that the increase in oxidative stress resulting from the infection induced an increase in antioxidant defenses but could not be reversed by it.

Additionally, the behavior of the TBARS versus TEAC correlations and TBARS versus URIC ACID was similar, suggesting that uric acid is an important component of the antioxidant defense of these animals or that IRS is associated with the infection. In this sense, the absence of correlation between these parameters found in the L-ARGININE group for both samples is further evidence of the absence of the IRS in animals in this group, probably as a result of vasodynamic effects attributable to NO.

Among the treatments, the only one that showed a significant correlation between TBARS and PARASITEMIA was dexamethasone (r = − 0.29, p = 0.026), which suggests that the selective inhibition of iNOS, associated with the anti-inflammatory potential of dexamethasone, decreases lipid peroxidation even with an increase in parasitemia. This suggestion is reinforced by the finding of negative correlations between TEAC versus PARASITEMIA and URIC ACID versus PARASITEMIA, since enzymatic antioxidant defenses and IRS suffer direct influence of lipid peroxidation. Additionally, Van der Steen et al.^44^ treated *P*. *berghei*-infected C57BL/6 mice with high doses of dexamethasone and blocked the development of pulmonary symptoms by those animals.

In this experimental model, considering that all animals were exposed to the same food supply, high values of uric acid indicate the existence of ischemia and reperfusion syndrome^45^, which may be caused by the decrease in the caliber of blood vessels, anemia, or obstruction of blood flow by the occurrence of cytoadherence.

A significant positive correlation was found for URIC ACID and TBARS levels in both samples and for both CONTROL and DEXAMETHASONE groups, suggesting that IRS arises from the increased oxidative stress in these animals as a consequence of disease progression and that NO synthesis may not exert an important effect in this case. On the other hand, for group L-ARGININE, in both samples the values of the coefficient of correlation approached zero, suggesting adequate blood supply to these tissues, possibly as a result of NO-attributable vasodilation.

Treatment with L-arginine did not promote any modification in the antioxidant capacity during the period studied. On the other hand, it significantly increased lipid peroxidation, but only on the first day of infection. The decrease in lipid peroxidation in subsequent days can be explained by the decrease in the IRS, justified by the low levels of uric acid for animals of this group during the entire period of infection.

The high correlations (moderate to strong) for TEAC versus URIC ACID in all groups arises from the simple fact that uric acid, by itself, is an antioxidant, in addition to being a marker of IRS. The same is true for the positive correlations between TEAC versus NN and NN versus URIC ACID, displayed by most of the groups.

Among the most unusual results, it is noteworthy the absence of differences in the levels of pulmonary nitrites and nitrates, independent of the use of inhibitor (dexamethasone) or stimulator of their synthesis (L-arginine). The possible explanations for such phenomena arising from compensatory physiological effects, such as vasoconstriction caused by NOS inhibition, seem to stimulate the production of mediators that cause vasodilation, such as acetylcholine and bradykinin, which are bronchoconstrictors. Conversely, it is possible that pulmonary hypertension on malaria, reported by Lacerda et al.^46^, as caused by the inhibition of NO by treatment with dexamethasone, along with the need for oxygen as a result of hemolysis, stimulates the synthesis of eNOS, which increases the expression of eNOS receptors in the lungs^47^.

The opposite effect occurred for the L-ARGININE group, in which an increase in nitrites and nitrates was expected, but despite the lack of statistical significance, the increase stood numerically below that of the other two groups. It is worth mentioning that after formation, L-arginine can follow two paths: the formation of ornithine and urea (action of arginase) or the formation of citrulline and NO (action of NOS). Additionally, interleukins (IL) 13 and 14 act over arginase, directing L-arginine to the synthesis of ornithine that is converted by the action of an aminotransferase to proline. This route has a fibrogenic role, since proline is an essential amino acid in collagen^48^. On the other hand, cytokines IFN-γ, TNF-α, and IL-12 optimize the formation of NO and citrulline from the action of iNOS over L-arginine^49^. Thus, it is likely that the excess of L-arginine, depending on the profile of cellular response stimulated, follows the arginase route, promoting the clearance of pulmonary nitrites and nitrates^48^, resulting in fibrinogen synthesis, in an attempt to revert pulmonary damage caused by oxidative stress.

Another possibility is that the vasodilation produced by excess NO increases the availability of O_2_, a substrate of NADPH oxidase, resulting in greater production of superoxide radicals and, consequently, peroxynitrite^8^. According to Wedgwood et al.^50^, peroxynitrite levels impose a negative feedback on NOS, *i*.*e*., the more peroxynitrite is synthesized, the greater the inhibition of NOS.

Additionally, the absence of differences between the groups for the values of pulmonary NN may be the result of the existence of a complex system of non-adrenergic non-cholinergic (NANC) neural fibers in the lungs of mammals, capable of producing large quantities of NO and, therefore, to masque NO levels arising from malaria in this tissue. This suggestion is reinforced by the absence of correlation between NN and TBARS levels in all groups for lung samples. In contrast, for brain samples, both groups POSITIVE CONTROL and DEXAMETHASONE showed significant positive correlations, while the L-ARGININE group showed no correlation between these parameters. These data suggest that, at least partially, oxidative stress associated with the development of the disease is derived from the production of NO, as pointed out by several authors, in addition to the participation of IRS^3^, which may have been reversed in the animals treated with L-arginine, due to its vasodilator effect.

### Cerebral findings

In the evaluation of brain oxidative parameters, an increase in lipid peroxidation was noted for mice treated with dexamethasone in relation to the other groups, mainly on the first day post-infection. Nevertheless, the opposite happens with the group of mice treated with L-arginine, where TBARS levels are significantly lower than the other groups.

The elevation of TBARS levels for the group treated with dexamethasone may result from a technical artifact, as brain tissue is rich in cholesterol and the drug may form cholesterol hydroperoxides, which may react with thiobarbituric acid, greatly increasing the absorbance of brain samples. A finding that may corroborate this statement is that the values of TEAC do not change in the first days of study for all groups. The possibility that lipid peroxidation occurs in this initial period by an increase in IRS was eliminated since uric acid values for this group of animals are similar to those of the other groups until the tenth day of infection.

Nevertheless, it seems that the oxidative effect of nitric oxide was overcome by its vasodilator effect, since the production of uric acid in mice treated with L-arginine was significantly lower when compared to other groups, notably from the 10th day of infection. However, the probable vasodilation presented by the L-ARGININE group caused no changes in the survival rate of these animals. Contrary to this, Ong et al.^51^ reported that L-arginine administration to mice with cerebral malaria improved blood flow and survival rate. This difference may be due to particularities in dose and administration protocols: whereas Ong et al.^51^ provided continuous delivery of L-arginine (10–200 mg kg^−1^) though subcutaneous osmotic pumping, in the present study L-arginine (120 mg kg^−1^) was administered only once a day though gavage.

The re-establishment of antioxidant capacity can be decisive for the survival of mice infected with *P*. *berghei*. The antioxidant capacity decreased significantly in all tested groups. However, only for the DEXAMETHASONE group this antioxidant capacity was significantly reversed from the 10th day, reinforcing the idea of Favre et al.^12^ and Maneerat et al.^13^ that the oxidative stress induced by nitric oxide in the cerebral microenvironment contributes to the severity of the disease.

Another point that deserves to be highlighted for the group treated with dexamethasone is that despite the inhibition of iNOS, there was only a significant increase in serum uric acid concentration from the 15th day, signaling that the beginning of IRS coincides with the starting point of death in this group. The finding of a positive correlation between TBARS and NN for the DEXAMETHASONE group corroborates this observation.

A factor that may have contributed significantly to the late start of the IRS in this group is the inhibition of the inflammatory process, which is necessary for the occurrence of cytoadherence^52^. Curiously, Goldring and Ramoshebi^53^ investigated the effect of dexamethasone on the cytoadherence of infected erythrocytes to monocytes and concluded that it can reduce cytoadherence, but this effect was also seen for other antioxidants studied, leading the authors to suggested that this effect may be due to indirect antioxidant properties of dexamethasone.

Furthermore, Schetters et al.^37^ claim that cerebral lesion in *Plasmodium*-infected mice is a consequence of immunological reactions, and dexamethasone is capable of inhibiting TNF-α production, but only before the transcriptional phase. Moreover, dexamethasone was also able to prevent brain hemorrhage and thermoregulation collapse in the mice model of the disease^54^.

Additionally, DEXAMETHASONE is the only group that displays a significant positive correlation between URIC ACID and PARASITEMIA, reinforcing the idea that IRS occurs on a temporal scale. The absence of correlation between NN and PARASITEMIA for all groups and both samples strongly suggests that NO levels do not influence the evolution of parasitemia.

Considering the different treatments administered, the more promising results were seen with the dexamethasone treatment, since animals exhibited a significantly higher survival rate and decreased progression of parasitemia when compared to the other groups. These data suggest that selective inhibition of iNOS, associated with the anti-inflammatory potential of dexamethasone, might decrease lipid peroxidation even with the increase in parasitemia.

In contrast, administration of L-arginine, regardless of not significant modification in NN concentrations, promoted vasodilation in both organs, proven by a decrease in the concentrations of uric acid, yet with no effect on the survival rate of these animals.

Nevertheless, the cerebral oxidative changes promoted by the administration of dexamethasone were somehow different from those presented by other groups. The re-establishment of the cerebral antioxidant capacity after the 10th day of infection is noteworthy, suggesting the participation of oxidative stress in the brain as a result of plasmodial infection, as well as the inhibition of brain NO synthesis, which promoted the survival rate of almost 90% of the animals until the 15th day of infection, with possible direct interference of ischemia and reperfusion syndrome, as seen by increased levels of uric acid.

Moreover, dexamethasone prevented or reduced the development of cerebral malaria in several experimental studies in rodents^25,54–56^ and non-human primates^57^, yet surprisingly, human clinical trials fail to prove beneficial effects for dexamethasone treatment of cerebral malaria^58,59^. Nevertheless, a systematic review on this subject suggested that the lack of positive effects may result of low number of subjects enrolled^60^. Indeed, Vandermosten et al.^61^ suggest that corticoid treatment for malarial complications is not well explored and may hold promise.

## Conclusion

Recently, the role of NO in the physiopathogenesis of malaria has been extensively studied. Nevertheless, its precise involvement in the underlying mechanisms of the disease is still controversial. The present study presents the inhibitory effects of dexamethasone on brain nitric oxide synthesis and its relationship to increased survival in a mouse model of malaria.

The data of the present study showed that brain iNOS inhibition by dexamethasone promoted an increase in the survival rate of *P*. *berghei*-infected animals until the point at which it compromised the functioning of the cerebral microcirculation. Indeed, iNOS inhibition by dexamethasone seems to have stimulated a series of redox and immunological effects that, if compensatory hyperstimulated, may be responsible for the onset of severe forms of malaria.

## Supplementary Information

Supplementary Information.

## Data Availability

Data and a full description of the methods and materials are available at the Zenodo repository at https://zenodo.org/record/4287690#.X7xDYGhKiM8.
