# Supplementary material for: Dexamethasone increased the survival rate in Plasmodium berghei-infected mice
Source: Sci Rep. 2021 Jan 29;11:2623. doi: 10.1038/s41598-021-82032-7 (PMC7846581; doi:10.1038/s41598-021-82032-7)
Supplement: Supplementary file 1 — Supplementary Information. [file 41598_2021_82032_MOESM1_ESM.docx]

Figure S1 – Correlation studies between Thiobarbituric Acid Reactive Substances (TBARS) and Parasitemia for pulmonary samples of *Plasmodium berghei*-infected Swiss mice. Animals were pre-treated and received a daily dose of DEXAMETHASONE, L-ARGININE, or PBS (CONTROL).

Figure S2 – Correlation studies between Thiobarbituric Acid Reactive Substances (TBARS) and Parasitemia for cerebral samples of *Plasmodium berghei*-infected Swiss mice. Animals were pre-treated and received a daily dose of DEXAMETHASONE, L-ARGININE, or PBS (CONTROL).

Figure S3 – Correlation studies between Thiobarbituric Acid Reactive Substances (TBARS) and Uric Acid for pulmonary samples of *Plasmodium berghei*-infected Swiss mice. Animals were pre-treated and received a daily dose of DEXAMETHASONE, L-ARGININE, or PBS (CONTROL).

Figure S4 – Correlation studies between Thiobarbituric Acid Reactive Substances (TBARS) and Uric Acid for cerebral samples of *Plasmodium berghei*-infected Swiss mice. Animals were pre-treated and received a daily dose of DEXAMETHASONE, L-ARGININE, or PBS (CONTROL).

Figure S5 – Correlation studies between Thiobarbituric Acid Reactive Substances (TBARS) and Nitrites and Nitrates for pulmonary samples of *Plasmodium berghei*-infected Swiss mice. Animals were pre-treated and received a daily dose of DEXAMETHASONE, L-ARGININE, or PBS (CONTROL).

Figure S6 – Correlation studies between Thiobarbituric Acid Reactive Substances (TBARS) and Nitrites and Nitrates for cerebral samples of *Plasmodium berghei*-infected Swiss mice. Animals were pre-treated and received a daily dose of DEXAMETHASONE, L-ARGININE, or PBS (CONTROL).

Figure S7 – Correlation studies between Thiobarbituric Acid Reactive Substances (TBARS) and Trolox Equivalent Antioxidant Capacity (TEAC) for pulmonary samples of *Plasmodium berghei*-infected Swiss mice. Animals were pre-treated and received a daily dose of DEXAMETHASONE, L-ARGININE, or PBS (CONTROL).

Figure S8 – Correlation studies between Thiobarbituric Acid Reactive Substances (TBARS) and Trolox Equivalent Antioxidant Capacity (TEAC) for cerebral samples of *Plasmodium berghei*-infected Swiss mice. Animals were pre-treated and received a daily dose of DEXAMETHASONE, L-ARGININE, or PBS (CONTROL).

Figure S9 – Correlation studies between Nitrites and Nitrates and Parasitemia for pulmonary samples of *Plasmodium berghei*-infected Swiss mice. Animals were pre-treated and received a daily dose of DEXAMETHASONE, L-ARGININE, or PBS (CONTROL).

Figure S10 – Correlation studies between Nitrites and Nitrates and Parasitemia for cerebral samples of *Plasmodium berghei*-infected Swiss mice. Animals were pre-treated and received a daily dose of DEXAMETHASONE, L-ARGININE, or PBS (CONTROL).

Figure S11 – Correlation studies between Trolox Equivalent Antioxidant Capacity (TEAC) and Nitrites and Nitrates for pulmonary samples of *Plasmodium berghei*-infected Swiss mice. Animals were pre-treated and received a daily dose of DEXAMETHASONE, L-ARGININE, or PBS (CONTROL).

Figure S12 – Correlation studies between Trolox Equivalent Antioxidant Capacity (TEAC) and Nitrites and Nitrates for cerebral samples of *Plasmodium berghei*-infected Swiss mice. Animals were pre-treated and received a daily dose of DEXAMETHASONE, L-ARGININE, or PBS (CONTROL).

Figure S13 – Correlation studies between Trolox Equivalent Antioxidant Capacity (TEAC) and Uric Acid for pulmonary samples of *Plasmodium berghei*-infected Swiss mice. Animals were pre-treated and received a daily dose of DEXAMETHASONE, L-ARGININE, or PBS (CONTROL).

Figure S14 – Correlation studies between Trolox Equivalent Antioxidant Capacity (TEAC) and Uric Acid for cerebral samples of *Plasmodium berghei*-infected Swiss mice. Animals were pre-treated and received a daily dose of DEXAMETHASONE, L-ARGININE, or PBS (CONTROL).

Figure S15 – Correlation studies between Trolox Equivalent Antioxidant Capacity (TEAC) and Parasitemia for pulmonary samples of *Plasmodium berghei*-infected Swiss mice. Animals were pre-treated and received a daily dose of DEXAMETHASONE, L-ARGININE, or PBS (CONTROL).

Figure S16 – Correlation studies between Trolox Equivalent Antioxidant Capacity (TEAC) and Parasitemia for cerebral samples of *Plasmodium berghei*-infected Swiss mice. Animals were pre-treated and received a daily dose of DEXAMETHASONE, L-ARGININE, or PBS (CONTROL).

Figure S17 – Correlation studies between Nitrites and Nitrates and Uric Acid for pulmonary samples of *Plasmodium berghei*-infected Swiss mice. Animals were pre-treated and received a daily dose of DEXAMETHASONE, L-ARGININE, or PBS (CONTROL).

Figure S18 – Correlation studies between Nitrites and Nitrates and Uric Acid for cerebral samples of *Plasmodium berghei*-infected Swiss mice. Animals were pre-treated and received a daily dose of DEXAMETHASONE, L-ARGININE, or PBS (CONTROL).

Figure S19 – Correlation studies between Uric Acid and Parasitemia for pulmonary samples of *Plasmodium berghei*-infected Swiss mice. Animals were pre-treated and received a daily dose of DEXAMETHASONE, L-ARGININE, or PBS (CONTROL).

Figure S20 – Correlation studies between Uric Acid and Parasitemia for cerebral samples of *Plasmodium berghei*-infected Swiss mice. Animals were pre-treated and received a daily dose of DEXAMETHASONE, L-ARGININE, or PBS (CONTROL).
